# Maison De Correction De Meinengen

**Published:** 1829-08

**Authors:** 


### MAISON DE CORRECTION DE MEINENGEN.

*Case of general Emphysema.* By Dr. Jahn. (*Magazin für die Gesammt. Heilkunde.*)

A prisoner, who for some time had laboured under a dropsical affection, which appeared to depend upon habits of intemperance and scanty diet, was chastised by one of the keepers. On the next day no traces of the punishment were observed, excepting a slight ecchymosis on the left lumbar region. The second day, the neck, face, and breasts began to swell, and the crepitus which was felt on touching the swollen parts left no doubt that air was effused under the cellular tissue. The patient made no complaint, and his breathing was natural. During the next night the swelling extended rapidly over the head, the trunk of the body, and the limbs. There was now great anxiety, violent cough, and a sensation of oppression in the chest, as though the lungs were compressed and pushed upwards. In the morning, the sense of suffocation was so great that the man was desirous of procuring some instrument with which he might make incisions into the skin for the purpose of giving exit to the air.

Dr. Jahn, on the following day, found the patient in a very dangerous condition. He was sitting up in bed, supported by nurses. The head, trunk, and extremities were at least double their natural size. The upper eyelids looked like bladders, and were as large as an apple. Even the eyeballs were emphysematous, and projecting from the orbits. The parts within the mouth were also tumefied with air. The scrotum was of an enormous size, and the penis as large as an arm. An oily sweat, of a peculiar appearance, covered the whole surface of the body, which sounded like a tambour if struck, and crepitated loudly when pressed with the hand. There was violent dyspnœa; respiration quick, sibilous, and performed with great difficulty, the patient being obliged to extend his neck, and grasp something near him. Froth issued from the mouth, and he coughed frequently. He was incapable of speaking, but his senses were undisturbed.

A trocar was plunged into the scrotum, and instantly a large quantity of inodorous gas escaped with great force and a whistling noise through the canula. The patient was immediately relieved, and in every part the swelling quickly diminished. He breathed easier and deeper, and began to joke at his previous appearance. He now stated that he had at first experienced a peculiar sense of compression in the lungs, which gradually increased; and then a violent constriction of the glottis; and, lastly, a sense of suffocation, as if there was no exit for the air contained in the chest.

As the puncture of the scrotum was insufficient to give vent entirely to the air contained in every part of the body, several others were made with the same instrument in the limbs, back, and chest. Air still, however, continued to be disengaged in the cellular tissue; but its accumulation was prevented by repeated punctures.

As soon as the state of the patient would permit, the thorax was attentively examined; but the most careful inspection could discover neither fracture nor depression of the ribs, nor laceration of the muscles. In fact, no perceptible lesion existed; besides which, the man had complained of no internal pain. He spoke only of a stiffness in the trunk of the body, which soon ceased.

He was put upon a strict antiphlogistic regimen, and the next day he was quite well. For ten days air continued to accumulate under the skin in small quantities, but moderate frictions caused it to disappear. On the twelfth day the legs were slightly œdematous, but this symptom was quickly removed by appropriate treatment.

